# The loss of βΙ spectrin alters synaptic size and composition in the *ja/ja* mouse

**DOI:** 10.3389/fnins.2024.1415115

**Published:** 2024-08-06

**Authors:** Michael C. Stankewich, Luanne L. Peters, Jon S. Morrow

**Affiliations:** ^1^Department of Pathology, Yale University School of Medicine, New Haven, CT, United States; ^2^The Jackson Laboratory, Bar Harbor, ME, United States; ^3^Department Molecular Cellular and Developmental Biology, Yale University, New Haven, CT, United States

**Keywords:** ankyrin, postsynaptic, cerebellum, NCAM, CaM kinase II, PSD95

## Abstract

**Introduction:**

Deletion or mutation of members of the spectrin gene family contributes to many neurologic and neuropsychiatric disorders. While each spectrinopathy may generate distinct neuropathology, the study of βΙ spectrin’s role (*Sptb*) in the brain has been hampered by the hematologic consequences of its loss.

**Methods:**

Jaundiced mice (*ja/ja*) that lack βΙ spectrin suffer a rapidly fatal hemolytic anemia. We have used exchange transfusion of newborn *ja/ja* mice to blunt their hemolytic pathology, enabling an examination of βΙ spectrin deficiency in the mature mouse brain by ultrastructural and biochemical analysis.

**Results:**

βΙ spectrin is widely utilized throughout the brain as the βΙΣ2 isoform; it appears by postnatal day 8, and concentrates in the CA1,3 region of the hippocampus, dentate gyrus, cerebellar granule layer, cortical layer 2, medial habenula, and ventral thalamus. It is present in a subset of dendrites and absent in white matter. Without βΙ spectrin there is a 20% reduction in postsynaptic density size in the granule layer of the cerebellum, a selective loss of ankyrinR in cerebellar granule neurons, and a reduction in the level of the postsynaptic adhesion molecule NCAM. While we find no substitution of another spectrin for βΙ at dendrites or synapses, there is curiously enhanced βΙV spectrin expression in the *ja/ja* brain.

**Discussion:**

βΙΣ2 spectrin appears to be essential for refining postsynaptic structures through interactions with ankyrinR and NCAM. We speculate that it may play additional roles yet to be discovered.

## 1 Introduction

Deficiencies or mutation in the alpha II spectrin gene *Sptan1* and in four of the five beta spectrin genes (*Sptbn1, Sptbn2, Sptbn4, Sptbn5*, respectively encoding beta II, beta III, beta IV, and beta V spectrin) all lead to various developmental, neurologic, neuropsychiatric, cognitive and sensory pathologies in both animal models and in humans (Ikeda et al., 2006; Cousin et al., 2021; Miazek et al., 2021; Morrow and Stankewich, 2021; Stankewich et al., 2022; Van de Vondel et al., 2022; Lorenzo et al., 2023; Morsy et al., 2023). Conversely, relatively less is known about the role of the erythrocyte type beta I spectrin (*Sptb*) in the brain [or in skeletal and cardiac muscle where it is also expressed (Winkelmann et al., 1990)]. Alternative transcription of *Sptb* generates two spectrin isoforms, βΙΣ1 and βΙΣ2, that differ only in their COOH-terminal sequence (Amin et al., 1993). βΙΣ1, the predominant form in red cells, has a shortened COOH-terminus that is poly-phosphorylated. It is unclear to what extent this isoform is expressed in brain. βΙΣ2 spectrin has a COOH-terminus that encompasses a pleckstrin homology (PH) domain that typically binds phosphatidylinositol phospholipids (PIP’s) (Lemmon, 2008). Earlier studies have established that βΙΣ2 spectrin is abundant at the postsynaptic density of cerebellar granule neurons, present on synaptic spines in the granule and molecular layers of the cerebellum, and found on a subset of intracellular vesicles that cluster in the neurite along microtubules (Malchiodi-Albedi et al., 1993). In the cortex, it is abundant in layer two in a distribution complementary to that of βΙΙΙ spectrin (Stankewich et al., 2010), and also is found on cortical interneurons in association with ankyrinR (Stevens et al., 2021). It is typically absent at the presynaptic membrane (Zagon et al., 1986; Lambert and Bennett, 1993; Malchiodi-Albedi et al., 1993; Nestor et al., 2011). As a group, the spectrins are a family of proteins that form organizing scaffolds for a bewildering array of ligands (De Matteis and Morrow, 2000). βΙ spectrin binds directly to both NCAM_180_ and NCAM_140_ (a process necessary for mediating neurite outgrowth) (Leshchyns'ka et al., 2003) and supports synaptic spine generation *in vitro* (Nestor et al., 2011). Its most fundamental interaction is with ankyrin, through which it participates in the organization and stabilization of a host of membrane channels and pumps (Stevens and Rasband, 2021). Yet surprisingly, no definitive neuropathology has been associated with *Sptb* loss or mutation, despite the identification of many human pedigrees with mutant βΙ spectrin and severe hemolytic disease (Zhang et al., 2001; Gallagher, 2004).

Considering that the profound anemia of βΙ spectrin deficiency might overshadow its direct neurologic consequences, we have sought to separate these events in animal models. Our laboratory has generated a genetically modified mouse model (Stabach, Stankewich, and Morrow, unpublished) with a floxed *Sptb* gene that has been used to explore specific questions relating to the role of beta spectrins on axonal node integrity (Liu et al., 2020a,b) and ankyrin mediated interneuron excitability (Stevens et al., 2021). However, as a first step to evaluating the global impact of *Sptb* loss as it occurs in a realistic setting accompanied by anemia during development, we have taken advantage of the jaundiced mouse (*ja/ja*). This mouse harbors a naturally occurring mutation in *Sptb* that replaces an arginine with a stop at codon 1,160, truncating the translated protein within spectrin’s ninth repeat unit (Bodine et al., 1984; Bloom et al., 1994). Homozygous *ja/ja* mice lack βI spectrin; the animals suffer a severe spherocytic hemolytic anemia that is 99% fatal by postnatal day six (Barker et al., 2000). By neonatal transfusion, the lifespan of these mice can be extended by several months (Kaysser et al., 1997), allowing an evaluation of the adult mouse brain that lacks βI spectrin but has developed under conditions of *in-utero* anemia. While the *ja/ja* mice managed in this way lack gross developmental deformities, their brains are typically a bit smaller and the angle of the brain to the spinal cord is steeper, a change attributed to the expanded marrow cavity in the skull (Kaysser et al., 1997). We were interested in any cellular or sub-cellular abnormalities in the brains of these naturally occurring βΙ spectrin-null mice. Four questions were addressed: (1) what structures in the brain utilize βI spectrin? (2) Do other β spectrins compensate? (3) Are there detectable morphologic or biochemical effects on synaptic structure or core postsynaptic density (PSD) proteins? (4) Why does the loss of βI spectrin not lead to a gross neurodevelopmental phenotype?

We find that there is an abrupt induction of βΙΣ2 spectrin expression in the brain beginning between postnatal days five to eight (p5-8). While the literature is conflicted, we find minimal expression of the erythrocyte isoform of spectrin (βΙΣ1) in the brain at any time, either pre- or postnatal. After its induction, βΙΣ2 spectrin is widely utilized throughout the brain, but most significantly in granule cells in the cerebellum, the CA1,3 region of the hippocampus, the dentate gyrus, cerebral cortical layer 2, the medial habenula, the ventral thalamus, and in several other nuclei. We detect no compensatory replacement by other spectrins in the absence of βΙ spectrin, at least not in the cerebellar granule cell dendrite and synapse. By ultrastructure analysis, there is a 20% reduction in the size of the βI spectrin-null granule cell synapses. The levels of the spectrin-binding proteins NCAM and ankyrinR are also reduced, but not the abundance of the core synaptic proteins PSD95 and CaM kinase IIα. Interestingly, βΙV spectrin levels increase in the *ja/ja* mouse, but do not appear as a replacement at the dendrite or synapse in the granule layer. The lack of a gross developmental phenotype accompanying the loss of βΙ spectrin mirrors the impact of *Sptbn2* deletion (which encodes βΙΙΙ spectrin) (Stankewich et al., 2010). These spectrins are both predominantly dendritic and postsynaptic, suggesting that their primary role is in synaptic maturation and stabilization, an ongoing process that occurs post-developmentally. For βΙΙΙ spectrin, one of its activities is guiding the maturation and elongation of synaptic spines (Stankewich et al., 2010; Gao et al., 2011). We conclude that βΙΣ2 spectrin is likely to play a role similar to that of βΙΙΙ spectrin by facilitating synaptic maturation for a subset of neurons. We anticipate that βΙΣ2 spectrin disruption will thus have functional consequences for cognitive processing and movement like the changes observed following βΙΙΙ spectrin disruption.

## 2 Methods

### 2.1 Mice

Jaundiced mice were maintained as heterozygotes (*ja/+*) on both the WBRe/J (WB) and C57BL/6 J (B6) strains of mice, and mating WB and B6 heterozygotes produced homozygotes (WBB6F1-*ja/ja* hybrids). Control mice were WBB6F1 hybrid littermates (*+/+ or ja/+*).

### 2.2 Animal husbandry

Mice were housed and cared for according to American Association for the Accreditation of Laboratory Animal Care (AAALAC) specifications at the Jackson Laboratories and in strict accordance with recommendations in the “Guide for the Care and Use of Laboratory Animals” of the National Institutes of Health. Protocols were in accord with the policies of the Jackson Laboratories Animal Use guidelines and approved by the Jackson Laboratories Animal Use Committee.

With respect to the ARRIVE 2.0 reporting guidelines: (1) Study design—the phenotype of homozygous *ja/ja* mice were compared with WT controls on the same genetic background; WT mice randomized with respect to gender were used to evaluate the developmental expression of βΙ spectrin in normal mice. (2) Sample size: twenty-two *ja/ja* mice were used, with an equal number of WT age matched controls. (3) Inclusion/exclusion criteria: not applicable. (4) Randomization: samples taken from mice within each group (*ja/ja*) vs. WT were evaluated in a blinded fashion. Obvious phenotypic differences between *ja/ja* and WT mice precluded randomization of the animals themselves. (5) Blinding/masking: samples harvested from animals were analyzed in a blinded fashion, and their origin revealed only following the analysis. (6) Outcome measures: changes in synapse size and protein composition in the brains of *ja/ja* vs. WT animals. (7) Statistical methods: paired *T*-test. (8) Experimental animals: *ja/ja* and WT animals were age matched irrespective of body weights (*ja/ja* were in general smaller). (9) Experimental procedure: RBC donors for transfusion of 1-day-old mice were adult B6*^+/+^* females or males. Recipients were newborn WBB6F1-*ja/ja* females or males. (10) Results: *ja/ja* animals lack βΙ spectrin, have diminished synaptic size (*p* = 0.037), and altered composition of three spectrin-binding proteins.

### 2.3 Hematologic parameters

Adult whole blood (~275 μL) was drawn from the retro-orbital sinus through EDTA-coated micro-hematocrit tubes directly into an Eppendorf tube containing 30 μL 20% EDTA in PBS (PBS; 10 mM NaCl, 155 mM KCl, 10 mM glucose, 1 mM MgCl_2_, 2.5 mM KHPO_4_, pH 7.4) and complete blood counts analyzed without dilution using an Advia 120 Multi-species whole blood analyzer (Bayer, Tarrytown, NY).

### 2.4 Transfusion protocols

RBC donors for transfusion of 1-day-old mice were adult B6-*+/+* females or males. Recipients were newborn WBB6F1-*ja/ja* females or males. Unaffected WBB6F1 littermates served as age-matched controls. Donor whole blood was drawn from the retro-orbital sinus into 6 heparin-coated microhematocrit tubes, expelled into PBS, spun down and suspended in 0.5 mL of PBS. Aliquots of 100 μL were injected into the superficial temporal vein of day old mice using a 30 g needle. Although transfused jaundiced mice still exhibited obvious signs of disease (Kaysser et al., 1997), transfusion allowed them to overcome the early crisis period and achieve a greatly increased life span.

### 2.5 Tissue procurement

Mouse brains for immunohistochemistry were obtained by rapid dissection after animals were fixed with 4% paraformaldehyde via trans-cardiac whole-body vascular perfusion. In this procedure animals were anesthetized without recovery with 1.2% Avertin, (0.2 mL/10 gm IP). The right atrium was nicked with scissors and perfused by syringe through the left ventricle. The blood was cleared from the animal by an initial bolus of phosphate buffered saline (PBS) ~20 mL prior to the flow of 4% paraformaldehyde ~50 mL. Good perfusion was judged by notable continuous flow of fixative exiting the atrium. Brains were removed and allowed to post fix for 24 h before paraffin embedding. Brains harvested for Western analysis were snap frozen and stored at –80°C.

### 2.6 Neuronal cell culture

Dissociated embryonic hippocampal and cortical neurons were isolated and cultured by standard methods (Wilcox et al., 1994). In brief, hippocampi were dissected from embryonic day 18.5 mouse brains and dissociated into individual cells by incubating in a trypsin-containing solution. The cells were then washed and plated on poly-L-lysine-coated (1 mg/mL) glass coverslips at a concentration of 150,000 cells per 35 mm dish in 1.5 mL neurobasal medium (Invitrogen) with 2% B27 supplement. Neurons were harvested at DIV 3, 6, 13, and 18 for Western blot analysis. After lysis in 20 mm HEPES, pH 6.9, 150 mm KCl, 2 mm MgCl_2_, 1 mm DTT, and 0.5% Triton X-100 and 10 min of centrifugation at 13,000 × g at 4°C, the supernatant was mixed with NuPAGE sample buffer (Invitrogen, catalog #NP0007) with 200 mM DTT, boiled for 5 min., and processed as below for brain homogenates.

### 2.7 Protein expression levels

Brains harvested from control and *ja/ja* mice were dounce homogenized 8 strokes in 5 mL extract buffer [20 mM Hepes. PH7.4, 120 mM NaCl, 25 mM KCl, 2 mM EDTA, 1 mM EGTA, 1% TX-100] supplemented with Protease Arrest (1:200) (Calbiochem). Homogenates were centrifuged 14 k RPM for 10 min. at 4°C. Supernatant was mixed with 2X NUPAGE sample buffer and run on NUPAGE Gel system according to the manufactures instructions (Invitrogen). Molecular weight standards were used to estimate the size of the protein bands reported in figures. βΙΣ2 spectrin (calculated MW 268091) ran at an estimated Mr. of 270 kDa (as did βΙΙ spectrin, which is similar in size); βΙΣ1 spectrin migrated as a 250 kDa protein (calculated MW 246261). After electrophoresis, proteins were electro eluted onto PVDF membrane and probed with primary antibodies for Western blot analysis. Protein bands were quantified using ImageJ, normalized to the intensity of actin run either on the same gel or on paired duplicate gels. Statistical analysis was performed using a one-tailed, two-sample unequal variance (heteroscedastic) *T*-test. Antibodies used in this study from StressGen were mouse anti-PSD95 (cat# VAM-PS002). Antibodies from Sigma were mouse anti-CaM kinase IIα (C265), mouse anti-tubulin clone TUB 2.1(T-4026), and mouse anti-actin clone AC-74 (A-5316). Mouse monoclonal anti-NCAM (clone 5B8) was from the Iowa hybridoma bank. Anti-clathrin was from Transduction labs #C43820; anti-NMDAR antibody was from Chemicon AB1516. Spectrin and ankyrin antibodies were anti-alpha II spectrin, clone AA6 (Millipore MAB1622), mouse monoclonal anti-βΙΙ spectrin clone 42/B (Pharmingen), rabbit polyclonal anti-βΙΙΙ spectrin (Stankewich et al., 1998), rabbit polyclonal anti-βΙV spectrin (a gift from Michela Solimena) (Berghs et al., 2000), mouse anti ankyrinB (RDI), rabbit anti ankyrinR (Cianci et al., 1988), a pan-reactive anti-spectrin αΙβΙ polyclonal antibody RASC (Cianci et al., 1988); a pan-reactive anti-βΙ spectrin monoclonal (VD4) (Yurchenco et al., 1982), and an anti-βΙΣ2 polyclonal antibody (βΙ C-term) that specifically recognizes the unique COOH-terminus of this isoform (Weed et al., 1996).

### 2.8 Immunolabeling

Immunostaining of brain paraffin sections was performed after antigen retrieval as before (Stankewich et al., 2006). Briefly, slides were de-paraffinized in xylene, hydrated in ethanol then PBS, and pressure-cooked at >100°C in a 0.1 M citrate buffer, pH 6.0. Nonspecific antibody binding was blocked with 2% BSA for 30 min at room temperature, followed by primary antibody incubation overnight at 4°C. All primary antibodies were diluted in 2% BSA/0.1% saponin PBS. *For immunofluorescence*, slides were incubated with either goat anti-mouse or anti-rabbit secondary antibody conjugated to Alexa dyes (Invitrogen) diluted 1:1,000 in the same buffer as used for the primary antibody for 1 h at room temperature. To stain nuclei slides were incubated in Hoechst dye for 10 min. Slides were visualized with an Olympus AX70 fluorescent microscope. Image acquisition was processed using OpenLab software (Improvision Inc., Lexington, MA). *For immunoperoxidase staining*, after primary antibody treatment as above, slides were incubated in ImmPRESS, (Vector Labs) anti-mouse or anti rabbit micro-polymer peroxidase reagent for 1 h at room temperature. They were then washed, post fixed for 15 min with 1% glutaraldehyde in PBS, washed, and incubated at RT in 0.1% diaminobenzidine, 0.01% hydrogen peroxidase in 50 mM Tris–HCL buffer, pH 7.4 for 15 min. Sections were briefly counterstained with hematoxylin and dehydrated in graded ethanol, cleared in xylene and cover-slipped with Cytoseal-60. Images were captured on a Qcolor 3 camera (Olympus).

In this study, the convention is followed for both Western blot and immunolabeling data that if a pan-reactive antibody to βΙ spectrin was used (RASC or VD4), the figure will be annotated as “βΙ.” If the anti-C-term antibody was used, the annotation will be βΙΣ2 reflecting the specificity of this antibody.

### 2.9 Electron microscopy

In coronal orientation the cerebellum was dissected into tissue slices approximately 1 mm thick and the full depth of cerebral layers. The slices were post fixed in 1% glutaraldehyde, 2% formaldehyde in 0.1 M Sodium cacodylate buffer, pH 7.4, and overnight at 4°C. The slices were then washed three times in 0.1 M sodium cacodylate over a one-hour period. They were then exposed to 1% osmium tetroxide in 0.1 M s-collidine buffer, pH 7.4, for 2 h on wet ice, and sequentially washed thrice in cold 0.1 M s-collidine buffer over 2 h. The slices were dehydrated in graded ethanol, passed through propylene oxide and infiltrated and embedded in Epox-812 resin. Semi-thin, (2 μm), sections were prepared on a Leica Ultracut ultramicrotome and stained with a mixture of AzureI/AzureII. Orientation was confirmed on the light microscope so that all layers of the cerebellum were properly retained. The blocks were trimmed, thin sectioned at 80 nm and stained with 2% uranyl acetate and lead citrate. Thin sections were viewed with a Carl Zeiss EM 910 electron microscope in low magnification mode (100–300×) to insure inclusion of all cerebellar layers. Purkinje cells were identified as a reference and the magnification was increased to 10,000×. Photomicrographs were taken in the granule layer postsynaptic densities in the immediate vicinity of the base of the Purkinje cell. The microscope was operated in hysteresis control conditions to avoid variation in magnification. Electron micrograph negatives, (image area 7.5cm^2^) were scanned on an AGFA model 1,400 negative scanner at 1,200 DPI, and contrast adjusted using Adobe Photoshop.

### 2.10 Ultrastructure analysis of PSD size

From 10 micrographs at 10,000 × magnification for each set in duplicate 200 PSDs were identified. Using the magic wand tool in Adobe Photoshop version 5.1, the boundary of each synapse was demarcated. Total pixel area of each synapse was recorded and plotted.

### 2.11 PSD preparation

PSD’s were prepared by differential centrifugation (Tandon et al., 1998; Phillips et al., 2001). Pre and postsynaptic fractions were distinguished based on their protein composition (Phillips et al., 2001). Briefly synaptosomes were prepared by a sucrose step gradient. Brain homogenates from 3–4 month WT mice were prepared in homogenate buffer (HB) (0.32 M sucrose, 0.1 mM CaCl_2_, 1 mM MgCl_2_,) were adjusted to 1.25 M sucrose with 2 M sucrose, 0.1 mM CaCl_2_. This homogenate was overlaid with 1.0 M sucrose, 0.1 mM CaCl_2_, and HB and then centrifuged at 100,000 g for 3 h at 4°C. After centrifugation, the membrane band at the 1.25/1.0 M sucrose interface was collected. Synaptosomal membranes were solubilized for 30 min. With 1% TX-100 at pH 6 and insoluble material was pelleted at 40,000 g for 30 min. The insoluble pellet was re-extracted in 1% TX-100 at pH 8.0 and pelleted. The pH 8.0 supernatant represents the presynaptic fraction; the pH 8.0 pellet contains proteins most associated with or in the PSD (Phillips et al., 2001).

### 2.12 mRNA analysis

Total RNA from whole mouse brain was obtained from a commercial source (Takara, San Jose, CA), cDNA was prepared with Superscript IV reverse transcriptase (ThermoFisher Scientific, Waltham, MA) and quantified with a Nanodrop spectrophotometer. Procedures followed reagent manufacturer’s directions for first-strand synthesis. Typically, 100 ng of RNA was used in each amplification. Isoform-specific PCR primers bridging consecutive exons were as follows:

For βΙΣ1 spectrin, the sense primer was: 5′-GGAAGTGTGCCAGTTCTCGAG-3′;

the antisense primer was: 5′-CGACTCCCAGGAACTAGACAAG-3′.

For βΙΣ2 spectrin, the sense primer was: 5′-AAGTGTGCCAGTTCTCGAG-3′,

the anti-sense primer was: 5′-CCTCTCATCCCCAACGGATTT-3′.

For qPCR, SYBR Green signal from every reaction at the end of each 60°C annealing extension step was recorded on a CFX96 Real Time System (BioRad). The presented data represent the mean values of quadruplicate determinations on three animals at each time point.

## 3 Results

### 3.1 βΙ spectrin expression is widespread in the mouse brain

**Figure 1:**
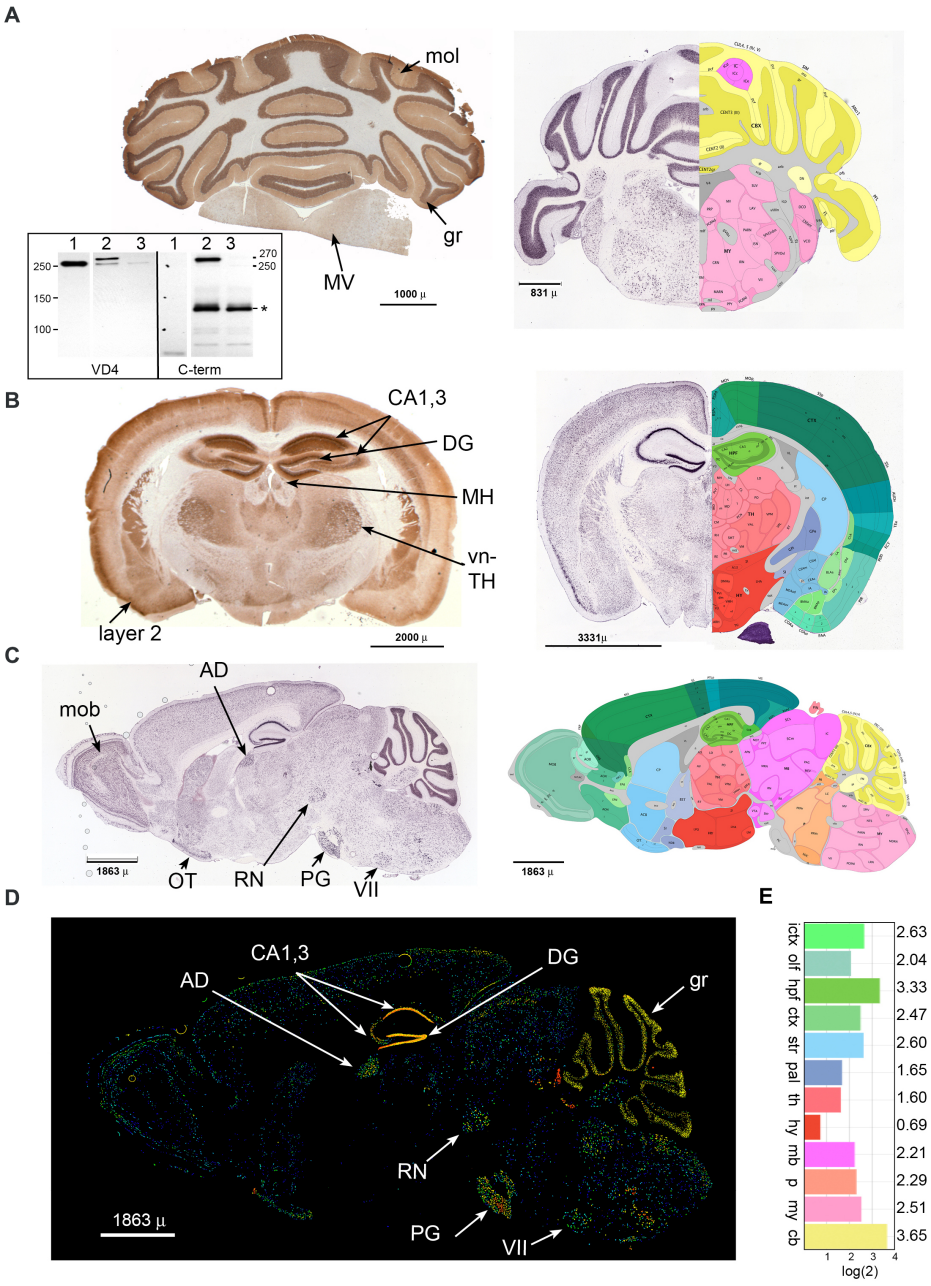
Beta 1 spectrin is widely utilized in the mouse brain. Immunohistochemical analysis for βΙ spectrin in coronal sections of the P60 mouse brain **(A,B)**, or *in situ* analysis of coronal and sagittal sections of the βΙΣ2 transcript in the P56 mouse (right panels in **A,B,** and figures **C,D**). *In situ* data is from the Allen Brain Atlas. **(A)** Coronal section of cerebellum. (Left) Immunohistochemical staining with the pan-reactive βΙ spectrin antibody VD4 demonstrating intense distribution in the granule layer (gr), and sparse but detectable staining in the molecular layer (mol) and the medial vestibular nucleus (MV). (Right) Composite image of *in situ* hybridization for βΙΣ2 spectrin (image 46 of 58, https://mouse.brain-map.org/experiment/show/75144624) juxtaposed with its best-matching diagram from the Brain Atlas. Areas of interest can be interrogated at the above URL. The intense hybridization in the granule layer and lessor signal in the molecular and MV and other areas is evident. (Inset) Western blot utilizing either VD4 or the C-term antibodies showing their specificity for βΙΣ1 vs. βΙΣ2 spectrin. Lane 1, RBC ghosts; lane 2, WT mouse brain synaptosomes; lane 3, mouse brain homogenate from our floxed *Sptb* mouse expressing nestin-Cre (that lacks βΙ spectrin in neuronal tissue) (Liu et al., 2020a). Note that VD4 detects βΙΣ1 spectrin (250 kDa) in RBC ghosts, both βΙΣ1 and βΙΣ2 spectrin (270 kDa) in synaptosomes, and trace amounts of βΙΣ1 spectrin in the nestin-Cre mouse brain. The C-term antibody detects nothing in RBC’s, the 270 kDa band of βΙΣ2 spectrin in synaptosomes, and no spectrin band in the nestin-cre brain. The C-term antibody also detects a non-specific band (*) at Mr. ≈ 120 kDa. The identify of this band, present only in brain homogenates, is unknown but does not appear to be related to βΙ spectrin based on its lack of reactivity with VD4 and persistence in the nestin-Cre sample. **(B)** Coronal section of brain. (Left) Immunohistochemical staining with the pan-reactive βΙ spectrin antibody VD4 demonstrating widespread expression. Positive areas include the hippocampus (CA1,3), dentate gyrus (DG), medial habenula (MH), ventral thalamus (vn-TH), and pyramidal cortical layer 2 (layer 2), along with widespread but less intense staining of other areas and nuclei. (Right) Composite image of *in situ* hybridization for βΙΣ2 spectrin (image 24 of 58, https://mouse.brain-map.org/experiment/show/75144624) juxtaposed with its best-matching diagram from the Brain Atlas. Areas of interest can be interrogated at the above URL. **(C)** Composite sagittal image of p56 mouse brain *in situ* hybridization for βΙΣ2 spectrin (image 15 of 19, https://mouse.brain-map.org/experiment/show/73834397) juxtaposed with its best-matching diagram from the Brain Atlas (image 15 of 21). Areas of interest can be interrogated at the above URL. Positive areas include the main olfactory bulb (mob), pyramidal olfactory tubercle (OT), anterolateral nucleus (AD), pontine gray (PG), red nucleus (RN), and facial motor nucleus (VII). **(D)** Sagittal section P56 brain expression of βΙΣ2 spectrin mRNA. Fluorescent image highlighting relative expression levels of this spectrin. This image is number 15 of 19, https://mouse.brain-map.org/experiment/show/73834397. Labeling is as above. **(E)** Relative levels of βΙΣ2 spectrin (*Sptb*) expression as detected by *in situ* hybridization from the dataset of the Allen Brain Atlas (atlas.brainmap.org., Spnb 34; 75144624). While hippocampus and cerebellum are the highest expressing regions, there is significant expression throughout most areas of the brain. The areas depicted (with the log(2) of their relative expression levels) are: ICTX, isocortex; OLF, olfactory; HPF, hippocampal formation; CTXsp., cortical subplate; STR, striatum; PAL, pallidum; TH, thalamus; HY, hypothalamus; MB, midbrain; P, pons; MY, medulla; CB, cerebellum.

Immune reactivity of βΙ spectrin in the brain has been noted in several reports (Riederer et al., 1986; Lambert and Bennett, 1993; Malchiodi-Albedi et al., 1993; Stevens et al., 2021). We have sought to refine these understandings by a more detailed immunological analysis using well-defined antibodies (Figure 1A, inset) to the now well-characterized isoform of βΙ spectrin (βIΣ2) found in the brain, augmented by a comparison to the *in-situ* expression analysis presented in the Allen mouse brain atlas (Allen Institute for Brain Science, 2004; Lein et al., 2007). We find that βΙΣ2 spectrin is more broadly expressed throughout the brain than previously appreciated. Coronal sections of the cerebellum and brainstem (Figure 1A) highlight the intense concentration of βΙΣ2 spectrin in the granule layer (gr) of the cerebellum, sparse abundance in the molecular layer (mol), and nearly total absence in white matter. The brain stem displays multiple regions of βΙΣ2 positivity such as the medial vestibular nucleus (MV). Coronal and sagittal sections of the brain (Figures 1B–D) reveal a dense concentration of βΙΣ2 spectrin in the CA1,3 regions and the dentate gyrus (DG) of the hippocampus. Other areas with abundant βΙΣ2 spectrin identified by both immunohistochemistry or *in situ* hybridization include the medial habenula (MH), ventral thalamus (vn-TH), cortical layer 2 (layer 2), red nucleus (RN), main olfactory bulb (mob), pyramidal layer of the olfactory tubercle (OT), anterodorsal nucleus (AD), pontine gray (PG), and facial motor nucleus (VII), as well as a diffuse distribution throughout much of the brain. As with the cerebellum, βΙ spectrin is absent in the white matter. This staining pattern was not discernably changed when the pan-reactive βΙ spectrin antibody (VD4) was used, indicating that in accord with the transcriptomics data βΙΣ2 spectrin is the major βΙ spectrin isoform expressed in adult mouse brain. The overall relative abundance of βΙΣ2 expression in cerebellum and hippocampus was 50–100% greater than any other region (Figure 1E).

### 3.2 βΙΣ2 spectrin co-localizes with postsynaptic PSD95 in culture

**Figure 2:**
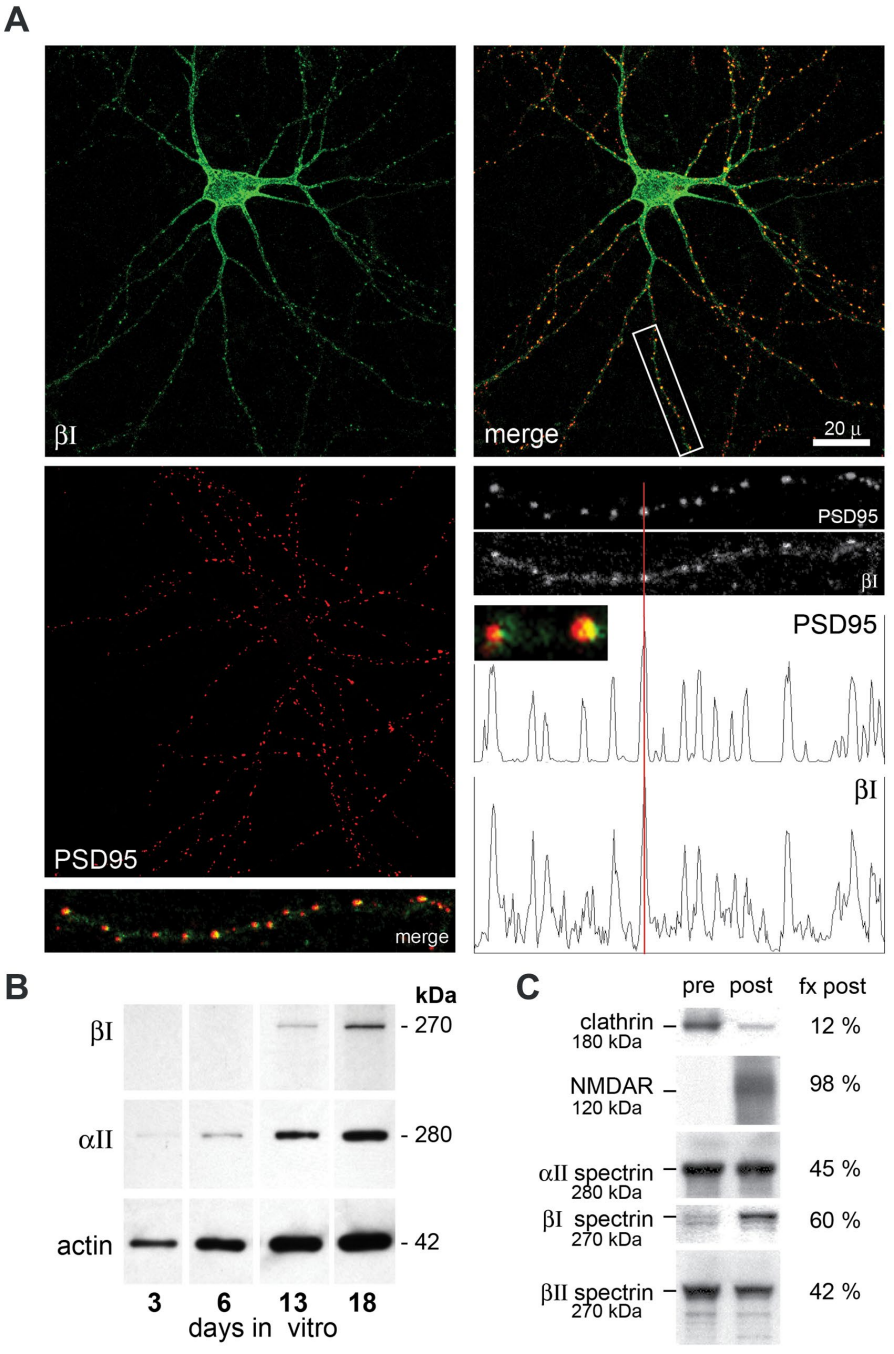
Hippocampal neurons assemble βΙ spectrin at nascent PSD’s. Hippocampal neurons from embryonic day 18.5 mice were cultured *in vitro*, then double immuno-stained with anti-βΙ-spectrin (antibody VD4, green) or anti-PSD95 (red). **(A)** DIV21 hippocampal neurons display abundant neurite processes and βΙ spectrin expression (green) throughout the cell. Staining with PSD95 reveals the formation of innumerable punctate areas along neurite processes marking nascent synapses. Merged images reveal a tight correspondence between βΙ spectrin and PSD95 staining at the neurite patches, as validated in the densitometry tracing along a neurite process (boxed area, enlarged 3×). Enlargement of individual puncta (small inset, enlarged 12×) shows that the PSD95 staining typically extends asymmetrically beyond the localized βΙ spectrin staining. **(B)** Expression of βΙ and αΙΙ spectrin and actin in E18.5 hippocampal neurons cultured *in vitro*. While αΙΙ spectrin and actin are constitutively expressed, βΙ spectrin as probed with the VD4 antibody is undetectable at 6 days *in vitro* (6DIV) but evident at DIV13. Based on its size (270 kDa), the spectrin appearing at 13DIV is βΙΣ2. **(C)** Analysis of synaptosomes from an adult WT mouse by density gradient separation allows the identification of (pre) and (post) synaptic fractions. The nearly complete separation by this method of clathrin (presynaptic) and NMDAR (postsynaptic) as analyzed by Western blotting and densitometry confirms the validity of the protocol (Phillips et al., 2001). In this analysis, βΙ spectrin at 270 kDa (blotted with RASC) is found predominantly (60%) in the postsynaptic fraction (fx post). Also visible in the presynaptic pool is a faint band at 250 kDa, presumably βΙΣ1 spectrin. βΙΙ spectrin is more abundant in the presynaptic fraction; αΙΙ spectrin is equally split between membrane fractions.

Embryonic hippocampal neurons mature *in vitro* after plating. Reflecting the sparse appearance of the βΙΣ1 spectrin isoform observed in embryonic and mature whole brain preparations (Riederer et al., 1986; Zimmer et al., 1992) cultured embryonic hippocampal neurons express only the 270 kDa βΙΣ2 isoform (Figure 2B). βΙ spectrin expression begins by DIV 13, and by DIV 21 distributes throughout the neurite processes where it concentrates intensely with PSD95 (Figure 2A). Based on analogy with βΙΙΙ spectrin (Efimova et al., 2017) and the disruptive effect βΙ spectrin constructs on spine formation (Nestor et al., 2011), βΙ spectrin appears likely to contribute to spine formation. Although our images lack sufficient resolution to make a definitive determination, extremely high-power views of each putative synapse (Figure 2A, small insert) suggests that while there is overlap of PSD95 and βΙ spectrin, a significant portion of the PSD95 extends asymmetrically beyond the spectrin immunostaining, raising the possibility that at least in cell culture βΙ spectrin may be in the spine but peripheral to core postsynaptic proteins. An association of βΙ spectrin with postsynaptic structures can also be confirmed biochemically in whole brain preparations after sedimentation gradient purification of isolated synapses (Figure 2C). Using clathrin and NMDAR as well documented markers of the presynaptic and postsynaptic compartments, respectively, (Phillips et al., 2001), we find that βΙ spectrin sorts predominately with postsynaptic isolates. βΙΙ spectrin is slightly more abundant in the presynaptic fraction, and αΙΙ spectrin, an obligatory partner for every beta spectrin, is more evenly distributed. These *in-vivo* results mirror closely the postsynaptic localization of βΙΣ2 spectrin observed by immunoelectron microscopy (Malchiodi-Albedi et al., 1993; Baines et al., 2001). While these studies do not reveal the precise substructure of spectrin at the PSD, they do demonstrate the facile and close association of this spectrin with nascent postsynaptic structures. The absence of any βΙ spectrin staining in the granule layer of *ja/ja* mice (see below) also indicates that the spectrin staining observed at the PSD cannot be due to cross-reaction with another protein (such as the non-specific band detected with the C-term antibody, Figure 1A inset).

### 3.3 The *ja/ja* mouse lacks βΙ spectrin but develops normally

**Figure 3:**
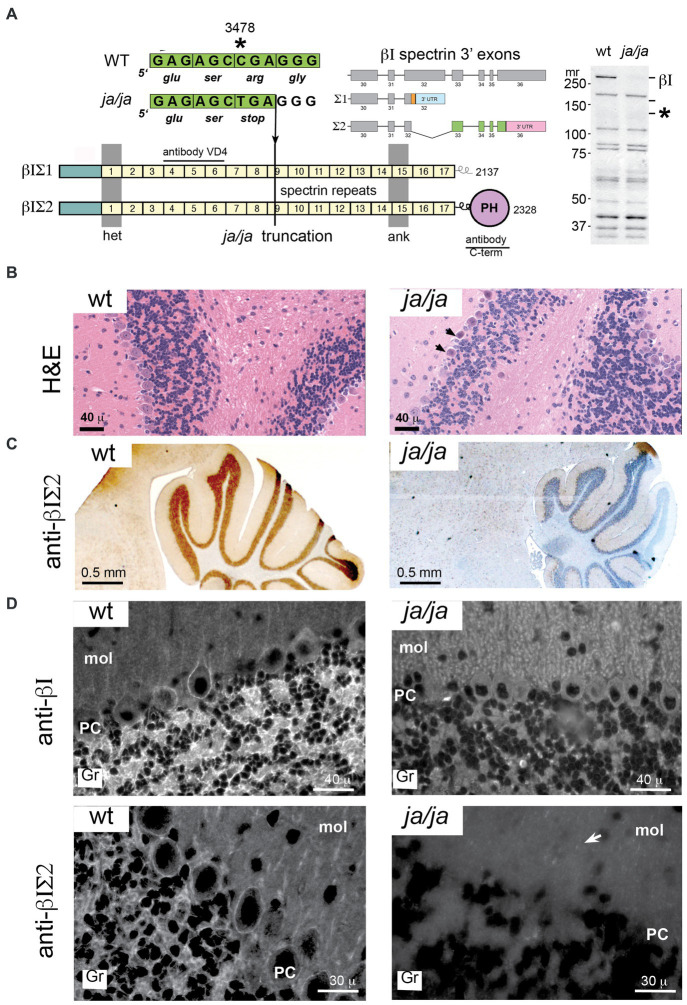
βΙ spectrin is not detected in the *ja/ja* mouse. **(A)** The *Sptb* gene is located on mouse chromosome 12. Two transcripts arise by alternative mRNA splicing from within exon 32 as depicted. This yields two protein products of Mr. 250 and 270, designated βΙΣΙ and βΙΣΙΙ respectively. These differ in their COOH terminus; βΙΣΙ displays a short poly-phosphorylated terminus and contains 2,137 amino acids, while the βΙΣΙΙ transcript encodes 2,328 residues and contains a classical pleckstrin homology domain (PH). In the *ja/ja* mouse there is a C - > T point mutation in exon 13 that generates a premature stop at nucleotide 3,478. This falls within spectrin repeat 9 (depicted by the numbered squares). Western blotting of a whole brain homogenate with RASC (a broadly pan-reactive polyclonal antibody that detects all transcripts of both αΙ and βΙ spectrin) demonstrates the absence of any full-length βΙ spectrin (βΙΣ2 at 270 kDa or βΙΣ1 at 250 kDa). These positions are labeled on the gel as βΙ. The mutated gene product if transcribed would yield a protein of 140 kDa. This position is marked by an asterisk (*). None of these proteins are detected in the *ja/ja* mouse brain. Also depicted are the epitope locations in βΙ spectrin of the monoclonal VD4 antibody and the C-term antibody used in this study. The VD4 recognizes both βΙΣ1 and βΙΣ2 spectrin; the C-term antibody is specific for βΙΣ2. **(B)** H&E stained sagittal sections of cerebellum from WT and *ja/ja* mouse brain. The *ja/ja* histology is largely intact, although scattered dark Purkinje cells that appear to be degenerating are noted (arrows). **(C)** Immunostains of sagittal cerebellar sections with C-term antibody reveals intense staining in the wild-type (WT) cerebellum granule layer and the complete absence of similar staining in the *ja/ja* mouse. **(D)** Immunofluorescent staining with the VD4 and C-term antibodies highlight βΙ spectrin’s abundance in the cerebellar granule cell layer (gr), its irregular staining in Purkinje soma (PC), and its scant labeling in the molecular layer (mol). βΙ spectrin staining with VD4 is completely absent in all areas in the *ja/ja* mouse, except for rare scattered red blood cells that are derived from the WT RBC transfusion that sustains these mice (two are evident in the image). The distribution of βΙΣ2 spectrin is coincident with the pattern detected by VD4, except there is no detection of residual RBC’s. The complete loss of staining in the granular layer in *ja/ja* mice with the C-term antibody excludes the possibility that the ≈120 kDa non-specific band this antibody detects in western blots (Figure 1A inset) could account for the synaptic staining. However, faint punctate staining in glial-rich areas can be detected in the molecular layer with the C-term antibody, even in the ja/ja mouse (arrow).

The *ja/ja* mouse was first described by Bernstein in 1959 (Bernstein and Russell, 1959) and termed *jaundiced* (gene symbol *ja*). Later it was determined that mice homozygous for the *ja* mutation have no detectable level of spectrin in their red cell membranes (Lux et al., 1979). Molecular genetic analyses found that the *ja* mutation maps to mouse Chromosome 12 and is caused by a C to T transition that produces a premature stop codon in exon 13 of the erythroid β spectrin gene (new gene symbol for jaundiced, *Sptb<ja>*) (Bloom et al., 1994) (Figure 3A). The *Sptb<ja>* transcript, if produced, would be truncated within spectrin protein repeat unit nine yielding a putative translation product of about 140 kDa. No such protein is detectable in the *ja/ja* mouse by immunoblotting with a pan-reactive spectrin αΙβΙ antibody (RASC) (Figure 3A).

Despite the absence of βΙ spectrin, the brains of *ja/ja* mice lack major morphological alterations. Histologically some Purkinje cells show occasional evidence of dark degeneration (Figure 3B, arrows). As expected, there is no βΙ spectrin immunostaining in the *ja/ja* mouse brain of either granule cells or the soma of Purkinje neurons with either C-term or VD4 antibodies (Figures 3C,D). The complete loss of staining in all areas with both βΙ reactive antibodies, areas rich in synapses, excludes the possibility that these antibodies are detecting an unrelated non-spectrin protein at the synapse.

### 3.4 Cerebellar granule cell synapses are altered in *ja/ja* mouse brains

**Figure 4:**
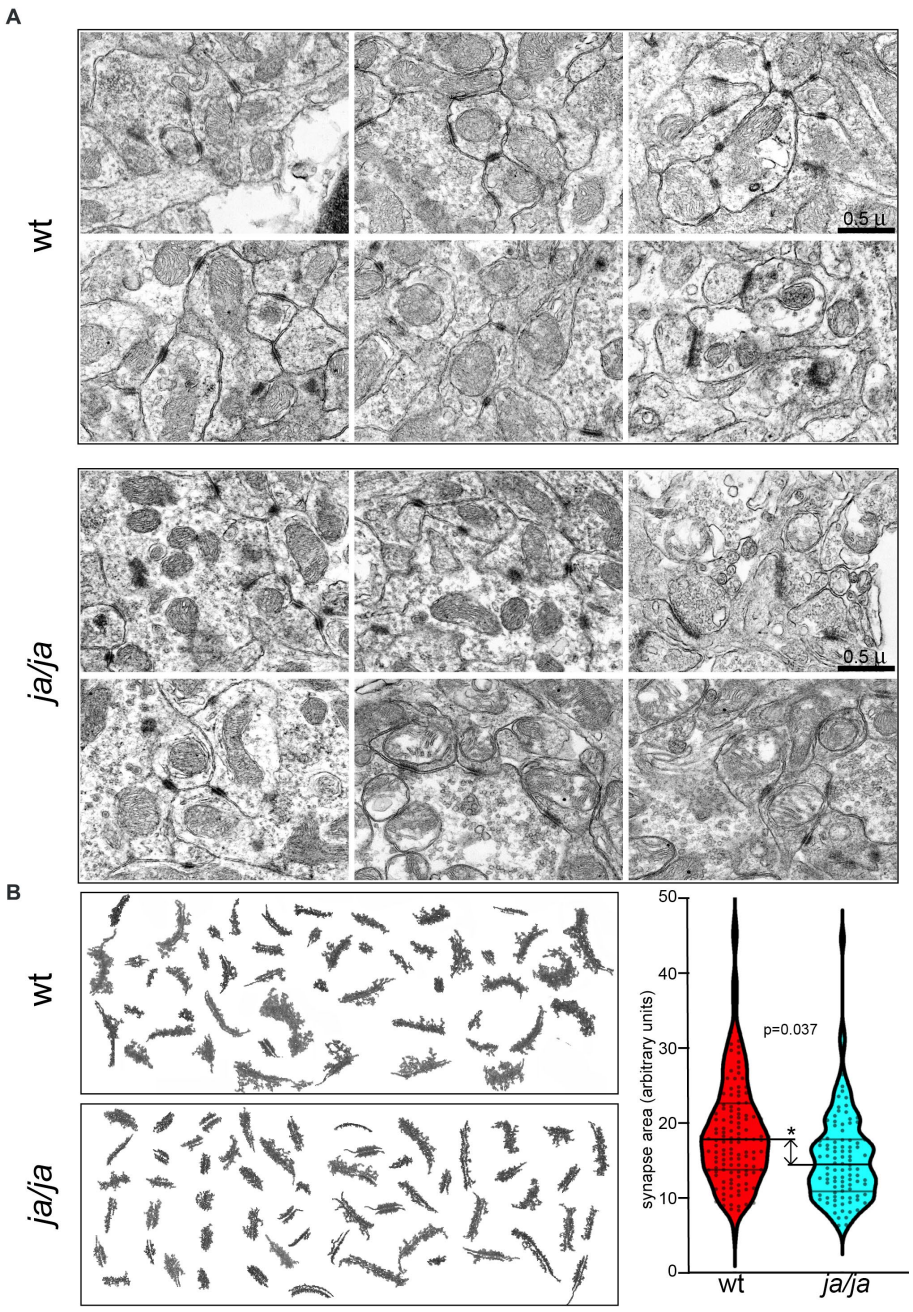
Synapse morphology is altered in *ja/ja* mouse brains. **(A)** Representative EM micrographs of epon-embedded thin sections of cerebellar granule layer from control WT and *ja/ja* mice at postnatal day 60 (p60). The synapses (dark stained structures) of the *ja/ja* mouse appear normal in number and overall distribution. **(B)** Compilation of synaptic membrane profiles from EM micrographs from two sets each of WT and ja/ja mice. Synapse size (pixel area) was quantified for 200 synaptic profiles from each set. The distribution of the relative PSD area between control and the *ja/ja* animals at p60 is graphed on the right panel. This difference is significant (*), the *ja/ja* synapses were on average 20% smaller (*p* = 0.037). Upper and lower cross bars represent ± SEM.

To study whether the absence of βΙ spectrin may correlate with structural abnormalities of PSDs, we analyzed ultra structural features of PSDs in the cerebellum. EM photomicrograph digital images were taken of the cerebellar granule layer adjacent to the base of the Purkinje cell, which served as a reliable landmark. From 10 micrographs at 10,000×, from two pairs of WT and *ja/ja* mice, the relative area of 200 randomly chosen PSD membrane profiles was compared. While no defects were noted in overall synapse abundance or organization (Figure 4A), the average surface area of PSDs in *ja/ja* brains was reduced by ~20% (*p* = 0.037, *n* = 200) (Figure 4B).

### 3.5 AnkyrinR, NCAM, and βIV spectrin levels are altered in *ja/ja* brains

**Figure 5:**
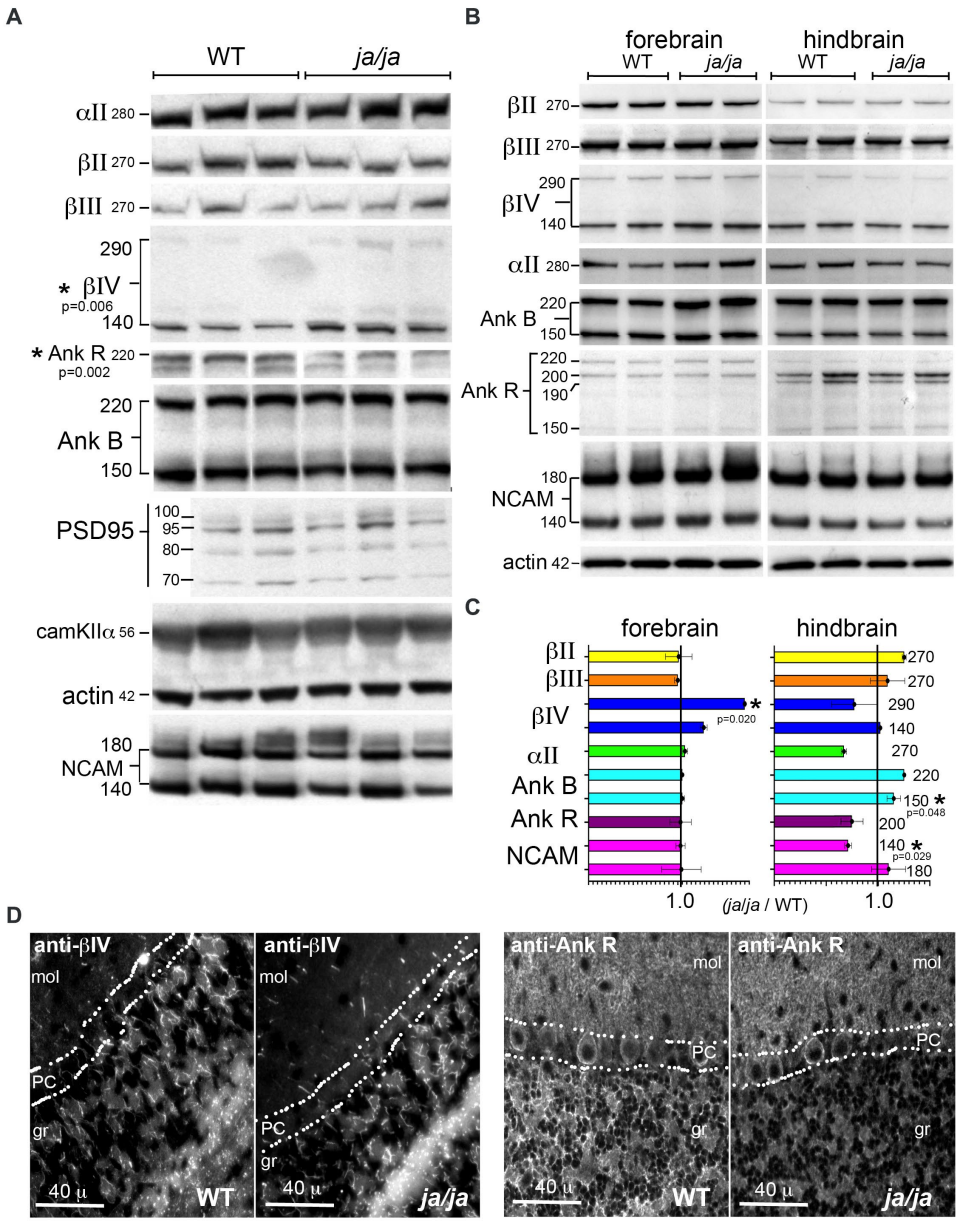
βΙ spectrin associated proteins are reduced in *ja/ja* mice. **(A)** Western analysis of WT and *ja/ja* mouse whole brain extracts (triplicate determinations) quantified by densitometry after normalization for actin. Asterisk marks the two proteins for which there was a significant change between the WT and *ja/ja*. βΙV spectrin increased by 63% in the *ja/ja* mice (*p* = 0.006), while the abundance of ankyrinR was reduced by 42% (*p* = 0.002). NCAM (both bands) trended lower (17%), but this change did not reach significance (*p* = 0.305). The level of the other proteins examined including the synaptic core proteins PSD95 and CaM kinase IIα were unchanged. **(B)** Western blot analysis of duplicate samples taken from either forebrain or hindbrain and analyzed by Western blotting and densitometry. All values were normalized to actin controls. The kDa of each band is as specified. **(C)** Abundance of proteins as determined by Western blots. As with the whole brain, values were normalized to an actin standard run on the same or companion gel and then compared as the ratio of the level in *ja/ja* vs. level in WT mice. Asterisk (*) marks the proteins (with their *p*-values, analyzed by 1 tailed, two-sample unequal variance (heteroscedastic) *T*-test) for which there was a significant change between WT and *ja/ja.* Error bars represent ±SEM. In the forebrain the level of βΙV_290_ spectrin, which was increased to 175% of the control (*p* = 0.020). The level of βΙV_140_ also trended toward an increase but did not achieve significance (*p* = 0.079). In the hindbrain, there was a 29% reduction in the level of NCAM_140_ (*p* = 0.029) and a 15% increase in ankyrinB_150_ (*p* = 0.048). The level of ankyrinB_220_ also appeared to increase, but this change was not significant (*p* = 0.108). The level of αΙΙ spectrin and ankyrinR both trended lower in the hindbrain but neither reached significance (*p* = 0.103 and 0.354, respectively). When taken together, the whole brain data and the forebrain data appear similar, with the most significant change being increased in βΙV spectrin in ja/ja mice. The trend in the hindbrain in ankyrinR together with the ankyrinR reduction in the whole brain suggests this change is also meaningful. **(D)** Immunofluorescence analysis in the cerebellum confirms extensive reduction of ankyrinR in the cerebellar granule layer (gr), but its preservation in the Purkinje cell soma (PC) and in the molecular layer (mol). The distribution of βΙV spectrin appears unchanged in the granule layer.

In the red cells of *ja/ja* mice, the levels of junctional components ankyrinR, band 4.1 and actin are largely unchanged and remain bound to the reticulocyte membrane (Bodine et al., 1984). In the brain, unlike red cells, multiple different beta spectrins are expressed, as well as multiple ankyrins. Of interest was the impact of βΙ spectrin loss on the levels of the other spectrins and their common ligands, as well as on the core components of the synapse. In Western blots of whole brain homogenates from adult mice (Figure 5A), most proteins examined remained unchanged in the *ja/ja* mice, except for a 63% increase in total βΙV spectrin (*p* = 0.006) and a 42% reduction in total ankyrinR (*p* = 0.002) (Figure 5A). NCAM_140_ also trended lower (17% reduction) in the *ja/ja* mice, but this change did not achieve significance (*p* = 0.305). Given that small changes in protein levels might be obscured by the abundance and widespread expression of these proteins throughout the brain, including regions largely devoid of βΙ spectrin, a regional variation in expression was sought by separately evaluating the forebrain and hindbrain (Figure 5B). As in the whole brain, the level of βΙV_290_ spectrin in the forebrain was increased to 175% of control (*p* = 0.020), while the hindbrain showed no change (*p* = 0.032). Both ankyrinR and αΙΙ spectrin trended lower in the hindbrain, but neither change achieved significance (*p* = 0.354 and *p* = 0.103 respectively). NCAM_140_ in the hindbrain was reduced to 71% of its WT value (*p* = 0.029). There was a 15% increase (*p* = 0.048) in ankyrinB_150_ in the hindbrain (Figure 5C).

The distribution of βΙV spectrin and ankyrinR in the cerebellum was also compared (Figure 5D). In this region, the pattern of βΙV staining, presumably confined to nodes and the initial axon segments of Purkinje cells, was unchanged in the *ja/ja* mice. Conversely, there was nearly complete loss of immunostaining for ankyrinR in the granule (gr) layer of *ja/ja* mice, but no change in the βΙΙΙ spectrin-rich synaptic region accompanying the Purkinje cell dendrites in the molecular layer (mol) (Figure 5D). This suggests that the trend to reduced ankyrinR observed in the hindbrain expression data is probably real, and correlates with the loss of βΙ spectrin in the granule layer. The lack of ankyrinR in this layer also makes it unlikely that another beta spectrin is compensating, since all beta spectrins preserve their ankyrin binding site (Stabach et al., 2009). Reduced beta spectrin should also correlate with reduced αΙΙ spectrin, perhaps contributing to the (albeit not reaching significance) trend to lower αΙΙ spectrin levels observed in the hindbrain data. While the core components of the synapse, PSD95 and CaM kinase IIα were unchanged, the reduction in NCAM in the hindbrain of the *ja/ja* mouse may account for the reduction in synapse size in the granule layer, as discussed below.

### 3.6 βΙΣ2 expression begins after postnatal day five

**Figure 6:**
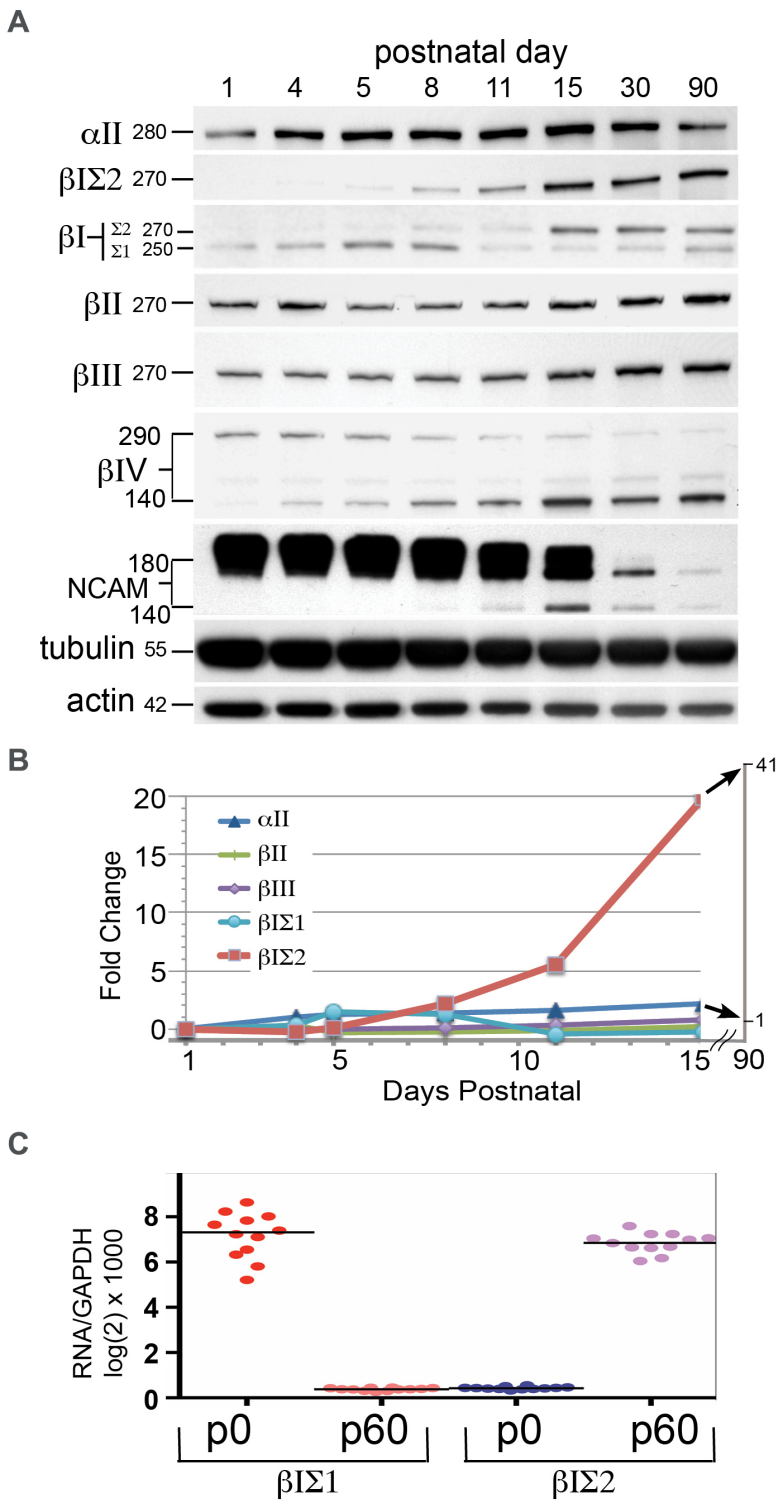
βΙΣ2 spectrin is not expressed before PD 6–8. **(A)** Time-course of protein appearance in developing postnatal WT mice, analyzed by Western blot. Each data point is a single animal. Blots were analyzed for βΙ spectrin with both VD4 and C-term antibodies. The size (kDa) of the protein bands are indicated. Note the significant rise in βΙΣ2 spectrin after p8 although a very faint band at 270 kDa can be detected in both the VD4 and C-term antibody blots at PD5. As βΙΣ2 expression grows, there is a precipitous and persistent drop in the levels of NCAM, along with switch to the 140 kDa isoform. **(B)** Quantitation of the postnatal changes in spectrin protein levels, measured as fold-change from levels at p1 after normalization to the actin control. **(C)** qRT-PCR measurement of βΙΣ1 vs. βΙΣ2 transcripts at postnatal day 0 (p0) and postnatal day 60 (p60). Quadruplicate measurements were performed on three animals at each age.

The loss of αΙΙ or βΙΙ spectrin is embryonic lethal and accompanied by severe developmental anomalies (Tang et al., 2003; Stankewich et al., 2011). The early postnatal lethality of the *ja/ja* mouse derives from its severe hemolytic anemia, but it was of interest to determine if changes in the brain due to an absence of βΙ spectrin might also contribute to the lethality of this genotype. The timing of βΙ spectrin expression in the developing brain suggests not (Figure 6). The expression of several spectrins and other synapse-related proteins were monitored by Western blots of whole wild-type mouse brain at the indicated postnatal ages (Figure 6A). To evaluate βΙ spectrin, the pan-reactive antibody (VD4) and the antibody specific for βΙΣ2 spectrin (βΙ C-term) were both utilized. While the levels of most spectrins were fairly constant over the time course of this experiment, there was a dramatic increase in βΙΣ2 spectrin. The band for this isoform was barely detectable at P5 in both the βΙ C-term blot and the VD4 blot (the band at 270 kDa). By P8 the βΙΣ2 isoform is clearly present. The increase in the βΙΣ2 level culminates in a more than 40-fold enhancement before leveling by p30-90 (Figure 6B). Conversely, the expression of the βΙΣ1 isoform peaks around P5-P8, and then falls precipitously to a persistent low-level of expression. Earlier studies have also observed this change in βΙ spectrin isoform expression during early postnatal development (Zimmer et al., 1992). This data might suggest that an isoform switch in βΙ spectrin appears around p6; the more relevant question is whether expression of βΙΣ1 spectrin persists in the maturing and adult mouse brain. It cannot be excluded that the low-level appearance of βΙΣ1 arises from unavoidable contamination of whole brain preparations by circulating reticulocyte RNA and red cells (that express enormous levels of βΙ spectrin relative to the brain). We also do not detect any βΙ spectrin expression in embryonic hippocampal neurons from E18.5 embryos until they have matured for at least 6 days in culture, after which they express βΙΣ2 spectrin (Figure 2). It is thus likely that βΙ spectrin is minimally utilized if at all in the developing brain, and only significantly appears when the βΙΣ2 isoform is induced during the period of active synaptogenesis.

Two other changes of interest are evident in Figure 6A: the isoform switch of βΙV spectrin from Mr. 290 kDa to Mr. 140 kDa that begins at p5-8, and the isoform switch (with down regulation) of NCAM from Mr. 180 kDa to Mr. 140 kDa that commences after p15. Isoform switching of both βΙV spectrin (Yoshimura et al., 2016) and NCAM (Sytnyk et al., 2006) have been previously associated with postnatal axonal and synaptic maturation. As postulated here, βΙΣ2 spectrin likely plays a similar role.

## 4 Discussion

Spectrinopathies, first recognized as a causal factor in several genetic anemia’s, are now appreciated to underlie many neurological disorders including ataxia, cognitive, neurosensory, psychiatric, developmental, and neurodegenerative syndromes (Cousin et al., 2021; Morrow and Stankewich, 2021; Stankewich et al., 2022; Van de Vondel et al., 2022; Lorenzo et al., 2023; Morsy et al., 2023). Experimental studies in mice and genetic linkage studies in human pedigrees support the importance of the spectrin-based framework for the maintenance of axonal and dendritic integrity. By virtue of its indirect and direct interactions with microtubules, actin filaments, adapter proteins (such as ankyrin), membrane proteins, and membrane lipids it plays a multifaceted role in directing the intracellular transport of cargo and provides a direct tether between the actin skeleton and receptors, transporters, and channels at regions of membrane specialization (Muresan et al., 2001; Leshchyns'ka et al., 2003; Stabach et al., 2008; Nestor et al., 2011; Ho et al., 2014; Machnicka et al., 2014).

Of the seven members of the spectrin gene family, the two most ubiquitous in the brain are *Sptan1* that encodes αΙΙ spectrin, and *Sptbn1* that encodes βΙΙ spectrin. Together these two spectrins coat the cell soma and neurite processes to form an axonal periodic actin-spectrin skeleton that is topographically and compositionally similar in many respects to the classical spectrin-actin skeleton of the red cell (Zhou et al., 2022). Defects in either of these spectrins may cause fatal developmental anomalies and severe cognitive deficits in surviving patients.

Conversely, deletion (or mutation) in *Sptbn2* that encodes βΙΙΙ spectrin does not cause gross developmental defects but does underlie several ataxias and cognitive deficiencies (Lise et al., 2012; Yıldız Bölükbaşı et al., 2017). βΙΙΙ spectrin is primarily confined to the postsynaptic side of dendrites and plays a critical role in spine maturation (Stankewich et al., 2010; Gao et al., 2011; Efimova et al., 2017). Many actin-binding proteins are enriched at the dendritic spine and have been elucidated as regulators of spine morphology (Matus et al., 2000). βIII spectrin is enriched at the base and neck of dendritic spines but is largely spared from the spine head in the PSD. In the absence of βIII spectrin, dendritic spines collapse and form aberrant shaft synapses (Efimova et al., 2017). Unlike αΙΙβΙΙ spectrin, βΙΙΙ spectrin does not appear to participate in the highly ordered neuronal periodic skeleton characteristic of the axon (Sidenstein et al., 2016). *Sptbn4* encodes βΙV spectrin, which plays a role in the organization of the initial axon segment and the nodes of Ranvier (Berghs et al., 2000). Defects in βΙV spectrin lead to ataxias and neuromotor deficits. *Sptbn5* encodes βV spectrin (Stabach and Morrow, 2000). This is a rare spectrin so far linked only to neurosensory deficits in the auditory pathway (Papal et al., 2013; Stankewich et al., 2022). Of the two spectrins found in erythrocytes, *Spta* and *Sptb*, encoding αΙ and βΙ spectrin respectively, only βΙ spectrin is expressed in the brain.

In the present study, four findings are significant:

- βΙ spectrin is more widely distributed throughout the brain than previously appreciated. While confirming the high abundance of this transcript and protein in the CA1,3 and dentate gyrus of the hippocampus and the granule layer of the cerebellum, it is apparent that many other nuclei and regions of the mouse brain utilize this spectrin. These include the medial habenula, ventral thalamus, cortical layer 2, red nucleus, and other areas. As with βΙΙΙ spectrin, βΙ spectrin is predominately confined to a somato-dendritic pattern, concentrating in dendritic spines and postsynaptic structures. There appears to be no overlap of βΙ vs. βΙΙΙ spectrin localization in any of the brain areas examined (Stankewich et al., 2010). Thus, all brain synapses can be categorized as either βΙ enriched or βΙΙΙ enriched; we believe it unlikely that both spectrins will be found co-localized in the same dendritic spine or synapse.
- The loss of βΙ spectrin does not inhibit synapse formation, but does diminish synaptic size, suggesting an impairment of synaptic maturation or organization.
- There is coincident reduction in the βΙ spectrin binding proteins NCAM and ankyrinR in the *ja/ja* mouse brain in the regions where βΙ spectrin is most utilized. There may also be a corresponding reduction in αΙΙ spectrin in these regions, although more data will be needed to confirm this observation. Ankyrin classically tethers spectrin to membrane channels, transporters, and receptors. This suggests that as with βΙΙΙ spectrin deletion (Stankewich et al., 2010), there will be specific deficits in the receptor or transporter composition of the βΙ spectrin-deficient synapses. It is interesting that while the loss of βΙ spectrin reduces ankyrinR in granule cells, the reverse is also true. Selective deletion of ankyrinR from granule cells reduces their βΙ spectrin (Peters et al., 1991). Similarly, NCAM anchors the presynaptic bouton to the postsynaptic density, and has been implicated in the formation and maintenance of PSDs (Korshunova et al., 2007). NCAM interacts directly with βΙ spectrin’s first structural repeat units (Leshchyns'ka et al., 2003). NCAM null mice have reduced synaptic associated βΙ spectrin, and phenocopy *ja/ja* mice in that their postsynaptic densities are also reduced in size by 20–30% (Sytnyk et al., 2006). The changes in NCAM and ankyrinR that accompany the loss of βΙ spectrin likely contribute to the diminished size and presumed instability of the *ja/ja* synapse.
- βΙΣ2 spectrin expression does not begin in the brain until after postnatal day 5, at which time it rapidly rises by over 40-fold to stabilize at adult levels by 30–90 postnatal days. While low levels of βΙΣ1 can be detected in the brain during embryonic development and in the postnatal period, contamination from circulating reticulocytes and red cells cannot be excluded as the source of these signals. Conversely, a low level of βΙΣ1 spectrin may continue to be utilized in the mature brain, e.g., possibly in the soma of PC’s that stain with the VD4 antibody, but are not consistently stained with either the βΙΣ2 specific antibody used in an earlier immunoEM study of rat brain (Malchiodi-Albedi et al., 1993) or with the C-term antibody used here. In this regard, it is interesting that the Allen Brain Atlas detects a very low level of βΙΣ1 message by *in situ* hybridization, and this signal is confined largely to a region encompassing the cerebellar PC layer (https://mouse.brain-map.org/experiment/show/73424302). Regardless, βΙ spectrin’s dominant role in the brain, similar to that for βΙΙΙ spectrin, appears to be limited to synaptic function rather than supporting larger scale morphologic development as do the αΙΙ and βΙΙ spectrins (Tang et al., 2003; Stankewich et al., 2011). The late postnatal appearance of the βΙΣ2 transcript in the brain presumably accounts for the lack of a detectable developmental phenotype in the *ja/ja* mouse.

The small increase in βΙV spectrin observed in the *ja/ja* brain is intriguing. We find no evidence that other β spectrins compensate for the loss of βΙ in dendrites or synapses, and the distribution of βIV is unchanged in the cerebellar granule layer. Yet, in other studies it has been observed that the loss of βΙV spectrin leads to an increase in βΙ spectrin, which appears to substitute for absent βΙV spectrin at the nodes of Ranvier (Liu et al., 2020a). That there is a reciprocal increase in βΙV spectrin accompanying the loss of βΙ spectrin suggests that βΙ spectrin-rich structures may exist natively for which βΙV spectrin can readily substitute. These putative structures, if they exist, are unlikely to be related to dendrite or synapse function, since βΙV spectrin does not appear there. The most likely candidate for such a structure would of course be the initial axon segments or nodes of a here-to-fore unidentified subset of neurons that utilize βΙ spectrin in lieu of βΙV spectrin. Given the broad distribution of βΙ spectrin throughout the brain (Figure 1), there are many candidates to consider. Alternatively, perhaps βΙ and βΙV spectrin both participate in other functions unrelated to their membrane structural roles. Possibilities would include their role in organizing intracellular compartments, organizing lipids, or modulating endocytosis or vesicular trafficking (Muresan et al., 2001; Stabach et al., 2008; Papal et al., 2013; Tseng et al., 2015).

A final question relates to whether βΙΣ2 spectrin is intimately involved with the core components at the PSD. In cultured cells, it appears in spines. However, the situation may be more nuanced *in vivo*. ImmunoEM demonstrates a close spatial association of βΙ spectrin with the PSD in rat brain (Malchiodi-Albedi et al., 1993). That study does not show staining in the PC soma, but as noted above, it is possible that the PC soma utilizes βΙΣ1 spectrin. Otherwise, the pattern of staining observed by that immunoEM study reflects the staining observed with VD4 and with the C-term antibody used here, all of which disappears in the *ja/ja* mouse. It thus seems unlikely that the βΙΣ2 specific antibody utilized in that immunoEM study was detecting a non-spectrin protein at the PSD.

## 5 Limitations

Transfusion of the *ja/ja* mouse has allowed identification of a neurological phenotype. An important caveat in these studies is the uncertain role of a premature and persistent anemia. The lifespan of these mice may also be insufficient to allow manifestation of all neurological or neurodegenerative consequences that follow βΙ spectrin deletion. These mice do not reach 1 year of age. By comparison, in the normoblastosis (*nb/nb*) mouse (ankyrinR deficient) maintained on the same hybrid genetic background as the *ja/ja* mice, Purkinje cell degeneration does not occur until 1 year of age (Peters et al., 1991). The appearance of a few degenerating Purkinje cells (Figure 3) hints that a similar outcome may befall older βΙ deficient animals, but that question cannot be definitively answered in the present study. Moreover, because transfused animals are still compromised by their anemia, studies to evaluate behaviors such as coordination, general activity, and learning are not feasible. However, we expect that functional defects in motor and cognitive function will manifest in older βΙ spectrin deficient animals.

## 6 Conclusion

We conclude that βΙΣ2 spectrin plays and important role in guiding dendritic spine and synapse maturation and presumably plays a role, acting through ankyrin and NCAM, in synapse function. Its loss results in reduced synapse size and reduction in two and possibly three constitutive spectrin-binding proteins associated with the dendritic spine and synapse (ankyrinR, NCAM, and possibly αΙΙ spectrin). Paradoxically, the loss of βΙ spectrin is accompanied by an increase in βΙV spectrin. In future studies utilizing the genetically engineered mouse models developed in our laboratory it will be important to determine the long-term consequences of global βΙ spectrin loss on behavioral, cognitive, and neurological function.

## Data availability statement

The raw data supporting the conclusions of this article will be made available by the authors, without undue reservation.

## Ethics statement

The animal study was approved by Jackson Laboratories Animal Use Committee. The study was conducted in accordance with the local legislation and institutional requirements.

## Author contributions

MS: Conceptualization, Data curation, Formal analysis, Investigation, Methodology, Writing – original draft, Writing – review & editing. LP: Investigation, Methodology, Writing – original draft, Conceptualization, Data curation, Funding acquisition, Project administration, Supervision, Writing – review & editing. JM: Conceptualization, Data curation, Formal analysis, Funding acquisition, Resources, Writing – original draft, Writing – review & editing, Project administration.
